# Tobacco and other risk factors for esophageal squamous cell carcinoma in Lilongwe Malawi: Results from the Lilongwe esophageal cancer case: Control study

**DOI:** 10.1371/journal.pgph.0000135

**Published:** 2022-06-15

**Authors:** Bongani Kaimila, Gift Mulima, Chifundo Kajombo, Ande Salima, Peter Nietschke, Natalie Pritchett, Yingxi Chen, Gwen Murphy, Sanford M. Dawsey, Satish Gopal, Kamija S. Phiri, Christian C. Abnet

**Affiliations:** 1 UNC Project, Department of Cancer Research, Lilongwe, Malawi; 2 Kamuzu Central Hospital, Department of Surgery, Lilongwe, Malawi; 3 St. Gabriel Hospital, Department of Medicine, Lilongwe, Malawi; 4 National Cancer Institute, Department of Cancer Epidemiology and Genetics, Metabolic Epidemiology Branch, Rockville, Maryland, United States of America; 5 Lineberger Comprehensive Cancer Center, University of North Carolina, Chapel Hill, North Carolina, United States of America; 6 Kamuzu University of Health Sciences, School of Public Health, Blantyre, Malawi; University of Cape Town, SOUTH AFRICA

## Abstract

**Objective:**

Esophageal cancer is the second commonest cancer in Malawi, and 95% of all cases are esophageal squamous cell carcinoma (ESCC). Very little is known about the epidemiology of ESCC in Malawi including risk factors. The main objective of the study was to evaluate and describe risk factors of ESCC in Malawi.

**Methods:**

We conducted a case-control study from 2017 to 2020 at two hospitals in Lilongwe, Malawi and consenting adults were eligible for inclusion. Endoscopy was conducted on all cases and biopsies were obtained for histological confirmation. Controls were selected from patients or their guardians in orthopedic, dental and ophthalmology wards and they were frequency matched by sex, age, and region of origin to cases. An electronic structured questionnaire was delivered by a trained interviewer. Multivariate conditional logistic regression models were used to assess the associations between subject characteristics, habits, and medical history and risk of ESCC.

**Results:**

During the study period, 300 cases and 300 controls were enrolled into the study. Median age of cases and controls was 56 years and 62% of the cases were male. Among cases, 30% were ever cigarette smokers as were 22% of controls. Smoking cigarettes had an adjusted odds ratio of 2.4 (95% CI 1.4–4.2 p = 0.003). HIV+ status was present in 11% of cases and 4% controls, which resulted in an adjusted odds ratio was 4.0 (95% CI 1.8–9.0 p = 0.001). Drinking hot tea was associated with an adjusted odd ratio of 2.9 (95% CI 1.3–6.3 p = 0.007). Mold on stored grain has an adjusted odd ratio of 1.6 (95% CI 1.1–2.5 p = 0.021).

**Conclusion:**

Reducing smoking cigarettes, consumption of scalding hot tea, and consumption of contaminated grain, could potentially help reduce the burden of ESCC in Malawi. Further investigation of the association between HIV status and ESCC are warranted.

## Introduction

Esophageal cancer (EC) is the sixth leading cause of cancer mortality worldwide, causing 500,000 deaths per year [1]. Squamous cell carcinoma and adenocarcinoma are the two main histologic subtypes, with esophageal squamous cell carcinoma (ESCC) accounting for 89% of all EC [2]. Globally, most ESCC occurs in geographical high-incidence areas in Central China, Northeastern Iran and Central Asia, and Africa [2–5]. Within Africa, there have been reports of high-incidence regions in southern, central and eastern Africa [6]. In Malawi, ESCC is the second most common cancer nationwide behind cervical carcinoma [7–9]. There were 1756 new cases and 1657 deaths in 2020, with an estimated 5-year prevalence of 2.45/100,000 population [9, 10]. Malawi has the highest reported age standardized rates for ESCC incidence and mortality in the world [10, 11]. Although ESCC has the highest incidence among cancers that are not HIV-associated, very little is known about the epidemiology of this cancer in this setting.

Tobacco smoking and alcoholic beverages are known causes of ESCC, but smoking and alcohol use are unlikely to explain high rates of ESCC in Malawi since the prevalence of these risk factors in the general population is low [7]. Many alternative causes for ESCC have been proposed, including dietary micronutrient and mineral deficiencies, indoor air pollution, poor oral health, socio-economic factors, mycotoxins, and hot beverages [12, 13]. However, the role these factors play in the etiology of ESCC in this setting is not clearly understood.

The primary objective of this study was to investigate risk factors for ESCC in Malawi through a case-control study implemented at a national teaching hospital and a regional hospital, specifically focused on the following exposures: tobacco smoking, alcoholic beverage consumption, HIV infection, scalding hot beverages and foods, family history of ESCC, cooking and heating practices, and potentially harmful dietary practices.

## Methods

The study was conducted at two hospitals in central Malawi, Kamuzu Central Hospital (KCH) and St. Gabriel Hospital (SGH) from 1^st^ August 2017 to 4^th^ April 2020. KCH is a public 1000 bed referral and teaching hospital in Lilongwe, central Malawi. It serves a population of 9 million people, half of Malawi’s population. Patients presenting to KCH come from Lilongwe district and referrals from all over central and northern Malawi. St. Gabriel Hospital (SGH) is a faith-based 250 bed semi-public hospital located 45km east of KCH in Lilongwe district which serves the surrounding districts. KCH and SGH are the only high-volume endoscopy centers in central and northern Malawi.

All adults 18 years old above presenting to the KCH and SGH endoscopy units with symptoms suggestive of ESCC and were screened for the study. Cases were enrolled prior to endoscopy to facilitate consent for research specimen collections during the procedure, but were then excluded if endoscopic biopsies did not confirm ESCC. Other exclusion criteria included being under 18 years old, being unwilling or mentally unable to give informed consent, being unable to give a medical history, or being deemed to be mentally unable to understand and comply with study procedures. During endoscopy, esophageal biopsies were taken from cases where possible for diagnostic confirmation by a board-certified pathologist at the KCH Pathology laboratory and to support molecular studies. None of the cases were surgical candidates, so cases with histologically confirmed esophageal malignancy were referred to the standard of care treatment, which included one or a combination of palliative chemotherapy, palliative esophageal self-expanding metal stent, or pain control.

Controls were frequency matched with cases on sex, 10-year age group and place of residence (district). The controls were selected from orthopedic, ophthalmology, and dental wards and were either patients or guardians (adult companions that typically come with all in-patient subjects). To be included in the study, controls had to be adults, to understand and be willing to consent to the study procedures, have no history of esophageal cancer, esophageal dysplasia, severe gastrointestinal disease or other cancers, and be able to provide a medical history. Six potential controls who were approached had to be excluded due to their medical history.

### Questionnaire data collection

After informed consent and study enrollment, cases and controls were interviewed using a structured questionnaire administered in the Chichewa language by study staff via a tablet, and the data was stored in a secure data facility. This questionnaire collected data on key variables including: smoking status (defined as smoking tobacco regularly for 6 months or more), alcohol use (defined as drinking alcohol regularly for 6 months or more), HIV status, tea temperature (defined as self-reported tea temperature classified as warm, hot, very hot and extremely hot), socioeconomic status score (SES score, defined as a composite score of owned household items (Radio, Diesel generator, TV, Paraffin lamp, Refrigerator, Mobile Phone, Iron, Car, Electricity, Bicycle) and education level), sibling history of esophageal cancer, fuel use (defined as the most commonly used household fuel) for cooking and for heating, mold on stored grain, and eating soil. The questionnaire was pre-coded whenever possible, and the collection program included logic checks to prevent miscoding. The questionnaire was derived from one used by similar studies of ESCC conducted within East Africa by the Africa Esophageal Cancer Consortium (AfrECC) [14, 15], with limited modifications to customize the exposures to our setting.

HIV status was obtained from participant medical records (health passports). For HIV negative participants, the result had to be less than 120 days from day of enrollment to be deemed a valid result. For all participants with invalid results or no HIV status recorded in records, an HIV testing facility was offered as per national guidelines. All participants refusing HIV testing were deemed as HIV ‘unknown’. There were no new HIV diagnoses during the study.

### Statistical analysis

We collated data into tables as counts and frequencies and compared groups using the Wilcoxon rank sum test, for continuous variables and using the Chi-square test for categorical variables. We then estimated the associations between exposures and ESCC risk using unconditional multiple logistic regression. We pre-specified our primary exposures of interest and selected the optimal parameterization based on logical collapsing of categories from the questionnaire and ensured that the final form of the variables had sufficient numbers for each value of interest. We iteratively selected potentially confounding variables based on known risk factors from other studies [16–22] and variables that meaningfully altered the beta estimates for our list of risk factors we aimed to explore. For the final model, case was the dependent variable in the model and smoking status, alcohol use, HIV status, tea temperature, social economic score, sibling history of esophageal cancer, solid fuel use, mold on stored grain, and eating soil were the independent variables. We had frequency-matched on sex, geographic region of residence, and age group so we adjusted for these variables. We report the Mantel-Haenszel pooled odds ratio and 95% confidence intervals from these models. We added a variable for hot nsima consumption about a third of the way through subject recruitment. We used a nested model with only those participants to test for an association with ESCC. In this nested model, other primary variables of interest were not materially altered. Finally, we tested the final model for goodness-of-fit using the Hosmer-Lemeshow test. All analyses were done in Stata (version 15, StataCorp).

This research project was conducted in accordance to the principles of good clinical practice and human subject’s protection. Written consent was obtained from the participants. If the participant was unable to write, a thumbprint was obtained together with written consent from a literate independent witness. Ethics approval was granted from the National Health Sciences Research Committee of Malawi protocol #16/7/1633 and the University of North Carolina at Chapel Hill Institutional Review Board protocol #LCCC-1608. NCI staff members had no access to personally identifiable variables or direct patient contact, so the NIH IRB deemed their individual participation to be not human subject research. The NCI staff’s participation was reviewed and approved by the NCI’s Division of Cancer Epidemiology and Genetics ethical review committee.

## Results

**Fig 1:**
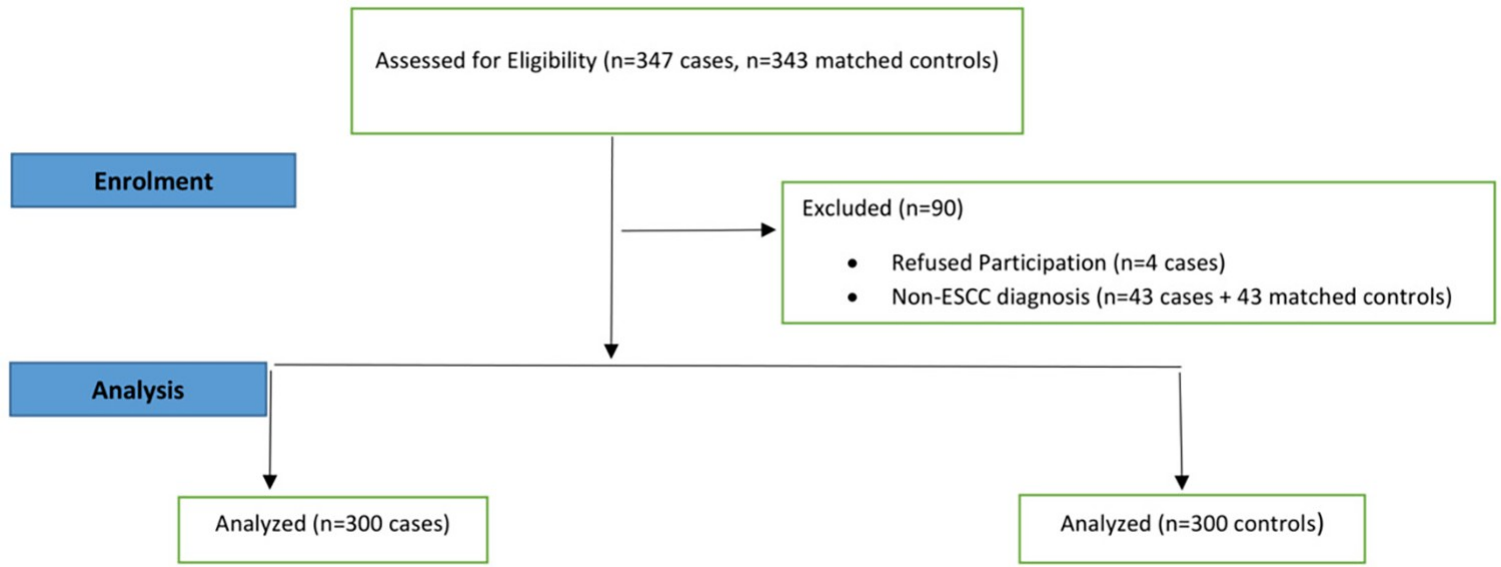
Participant enrolment flow into the ESCC case-control study.

We screened 347 potential cases and enrolled 343 cases and 343 matched controls. Four potential cases refused participation due to unwillingness to provide biospecimen. We removed 43 cases (due to a non-ESCC diagnosis after pathology) and 43 controls. The final analysis was therefore done among 300 case-control pairs. 86% (n = 259) cases had a histological confirmation of ESCC while 14% (n = 41) cases had a clinical diagnosis of ESCC. Participant flow is presented in **Fig 1**.

General characteristics of the 300 cases and 300 controls are presented in **Table 1**. The median age of both groups was 56 years and there were no significant differences in sex or geographic region of origin due to the matching protocol. 74% (n = 223) of cases and 75% (n = 226) of controls self-reported as being married while 69% (n = 207) of cases and 77% (n = 231) of controls self-reported being of the Chewa tribe. We present the adjusted odds ratios (aOR) and 95% confidence intervals (95% CI) from a single multivariable model for the pre-specified primary exposures in **Table 2**. The Hosmer-Lemeshow test showed a good fit for the final logistic model (P  =  0.26).

**Table 1:** Characteristics of participants enrolled in an ESCC case-control study in Lilongwe, Malawi.

		Cases, n = 300	Controls, n = 300	p-value*
Age in years, Median (range)		56 (19–92)	57 (19–90)	0.956^β^
Male, n (%)		187 (62%)	176 (59%)	0.358^α^
Region of Origin, n (%)				0.214 ^α^
	Northern	9 (3%)	3 (1%)	
	Central	279 (93%)	284 (95%)	
	Southern	12 (4%)	13 (4%)	
Time taken to travel to facility in hours, median (range)		1.5 (0.15–11)	1.5 (0.3–6)	0.417^β^
Occupation: Farmer, n (%)		198 (66%)	199(66%)	0.236 ^α^
Education, n (%)				0.078 ^α^
	None	83 (29%)	67 (22%)	
	Primary school	113 (37%)	105 (35%)	
	Secondary School	72 (24%)	87 (28%)	
	Tertiary	32 (10%)	42 (15%)	
Religion, n (%)				0.186 ^α^
	Christian	236 (78%)	243 (81%)	
	Muslim	32 (11%)	20 (7%)	
	Other	20 (7%)	29 (9%)	
	None	12 (4%)	8 (3%)	
Marital Status, n (%)				0.022 ^α^
	Single	14 (5%)	30 (10%)	
	Married	223 (74%)	226 (75%)	
	Divorced	9 (3%)	11 (4%)	
	Widowed	51 (17%)	30 (10%)	
	Separated	3 (1%)	3 (1%)	
Ethnicity, n (%)				0.019 ^α^
	Chewa	207 (69%)	231 (77%)	
	Ngoni	41 (14%)	33 (11%)	
	Yao	30 (10%)	12 (4%)	
	Other	22 (7%)	24 (8%)	

* Statistical Test used

α chi-square test

β Wilcoxon rank sum test

**Table 2:** Adjusted odds for ESCC for selected risk factors among participants enrolled in an ESCC case-control study in Lilongwe, Malawi.

		Cases n (%)	Controls n (%)	Unadjusted OR^α^ (95% CI)	Adjusted OR^β^ (95% CI)	P Value^β^
Smoking						
	Never Used Tobacco^ⱡ^	211 (70%)	235	Reference	Reference	-
	Self-Rolled Cigarettes only	17 (6%)	7	2.8 (1.1–7.0)	4.6 (1.6–13.7)	0.005
	Shop Bought Cigarettes only	29 (10%)	32	1.0 (0.6–1.8)	1.3 (0.7–2.7)	0.438
	Self-Rolled & Shop-Bought	30 (10%)	11	3.1 (1.5–6.5)	4.6 (1.9–11.1)	0.001
	Other Forms of Tobacco	13 (4%)	15	1.0 (0.4–2.1)	2.6(010–7.0)	0.059
Alcohol						
	Never Alcohol^ⱡ^	206 (68%)	210 (70%)	Reference	Reference	-
	Drank Beer only	24 (8%)	34 (11%)	0.7 (0.4–1.2)	0.5 (0.2–0.9)	0.033
	Drank Spirits only	8 (3%)	13 (4%)	0.6 (0.2–1.5)	0.4 (0.1–1.2)	0.110
	Drank Beer and Spirits	50 (17%)	35 (12%)	1.3 (0.8–2.2)	0.9 (0.5–1.7)	0.722
	Drank other forms of alcohol	12 (4%)	8 (3%)	1.4 (0.6–3.6)	0.9 (0.3–2.8)	0.922
Self-Reported Tea Temperature						
	Warm^ⱡ^	32 (11%)	50 (17%)	Reference	Reference	-
	Extremely Hot	14 (5%)	10 (3%)	2.1 (0.8–5.9)	2.3 (0.7–7.5)	0.170
	Very Hot	95 (32%)	114 (38%)	1.3 (0.6–2.5)	1.7 (0.8–3.7)	0.185
	Hot	142 (47%)	98 (33%)	2.3 (1.2–4.4)	2.9 (1.3–6.3)	0.007
	Never drank tea	17 (5%)	28 (9%)	1.0 (0.5–2.1)	1.3 (0.5–3.5)	0.657
Mold on stored grain						
	No^ⱡ^	169 (57%)	203 (68%)	Reference	Reference	-
	Yes	100 (33%)	71 (24%)	1.7 (1.2–2.5)	1.6 (1.1–2.5)	0.021
	Unknown	31 (10%)	26 (8%)	1.4 (0.8–2.6)	1.3 (0.7–2.4)	0.392
Ate Soil						
	Never^ⱡ^	193 (64%)	225 (75%)	Reference	Reference	
	Yes	105 (35%)	71 (24%)	2.1 (1.4–3.1)	1.8 (1.2–2.8)	0.008
	Unknown	2 (1%)	4 (1%)	0.6 (0.1–3.4)	0.2 (0.03–1.6)	0.136
Fuel for cooking						
	Firewood^ⱡ^	221 (74%)	230 (76%)	Reference	Reference	-
	Charcoal	64 (21%)	66 (22%)	0.3 (0.0–3.0)	1.00 (0.6–1.5)	0.898
	Electricity	14 (4%)	1 (1%)	14.5 (1.9–111.6)	15.4 (1.6–145.6)	0.014
	Other	1 (1%)	3 (1%)	1.0 (0.7–1.5)	0.2 (0.02–2.8)	0.238
Fuel for Heating						
	No Heating ^ⱡ^	198 (66%)	179 (60%)	Reference	Reference	
	Solid Fuel	98 (33%)	101 (33%)	0.9 (0.6–1.2)	0.7 (0.5–1.0)	0.059
	Non-Solid Fuel	4 (1%)	20 (7%)	0.2 (0.1–0.5)	0.2 (0.1–0.6)	0.006
SES Score (Mean [SD])		-0.7 (-1.9–0.5)	0.7 (-0.2–1.6)	1.0 (1.0–1.0)	1.0 (1.0–1.0)	0.635
HIV Status						
	Negative^ⱡ^	162 (54%)	195 (65%)	Reference	Reference	
	Positive	33 (11%)	12 (4%)	3.5 (1.7–7.0)	4.2 (1.9–9.4)	0.000
	Unknown	105 (35%)	93 (31%)	1.4 (1.0–1.9)	1.2 (0.8–1.8)	0.345
Sibling Died of ESCC						
	No^ⱡ^	269 (90%)	265 (89%)	Reference	Reference	-
	Yes	24 (8%)	10 (3%)	2.4 (1.1–5.1)	2.5 (1.0–5.9)	0.043
	Unknown	7 (2%)	25 (8%)	0.3 (0.1–0.6)	0.4 (0.2–1.0)	0.061

ⱡ Reference category

α Adjusted for, age, gender, and region of origin

^β^ Adjusted for age, gender, region of origin, smoking, alcohol, self-reported tea temperature, mold on stored grain, ate soil, fuel for cooking, fuel for heating, SES score, HIV status, sibling died of ESCC

Among ESCC cases, 30% (n = 89) compared with 22% (n = 65) of controls reported ever using tobacco for over 6 months with a median consumption of 6.2 pack years (range 0–75 pack years). Smoking status was associated with ESCC (aOR 2.5, 95% CI 1.4–4.2). Smoking self-rolled cigarettes only (aOR 4.6, 95% CI:1.6–13.7) or smoking both self-rolled and shop-bought cigarettes (aOR 4.6, 95% CI:1.9–11.1) were associated with ESCC, but smoking shop-bought cigarettes only was not (aOR 1.3, 95% CI: 0.7–2.7). Among ESCC cases, 31% (n = 94) compared with 30% (n = 90) of controls ever drank alcohol regularly for 6 months or more. Alcohol consumption was not associated with ESCC (aOR 0.7, 95% CI 0.4–1.1). Only 3% (n = 10) of cases and 3% (n = 9) of controls reported drinking more than 3 drinks per day. The number of drinks per day was not associated with ESCC. In addition, 4% (n = 11) of cases and 6% (n = 17) of controls reported to have ever used marijuana, and the aOR for marijuana use was 0.44 (95% CI 0.17–1.15).

Many previous reports have suggested that smoking tobacco and drinking alcohol synergistically increase the risk of ESCC. We explored that phenomenon here, fitting our model among smokers only to assess changes in risk by alcohol consumption. While we found that drinking alcohol did not significantly increase the ESCC risk associated with smoking, the risk estimates were non-significantly higher among drinkers. Smoking cigarette estimates among drinkers had an aOR of 10.7 (95% CI 2.5–45.2) for smoking self-rolled cigarettes only, an aOR of 5.5 (95% CI 1.9–15.8) for smoking shop bought cigarettes only, and an aOR of 9.8 (95% CI 2.8–33.0) for smoking both self-rolled and shop bought cigarettes.

Tea is a primary beverage in Malawi and most subjects regularly consumed tea. Self-reported tea temperature of “hot” was associated with significantly increased risk of ESCC, but there was no gradient in risk from “hot” to “very hot” to “extremely hot”. Nsima is the locally corn meal staple food and commonly consumed very hot. We observed that those who reported eating hot nsima had significantly higher risk for ESCC (aOR 2.8, 95% CI: 1.3–5.8). We also collected data on several other dietary practices that have previously been hypothesized to place subjects at higher risk for ESCC. Consumption of grain containing visible mold was reported by 33% (n = 98) of cases and 24% (n = 71) of controls (aOR = 1.6, 95% CI:1.1–2.5), and 35% (n = 105) of cases and 24% (n = 71) of controls reported regular consumption of soil (aOR = 1.8, 95% CI: 1.2–2.8).

Cooking outside in open spaces was reported by 64% (n = 191) of cases and 63% (n = 190) of controls. The majority of subjects reported using firewood as the primary cooking fuel and the primary heating fuel. A larger percentage of cases compared to controls reported use of electricity for cooking, but the total numbers using this fuel were small, so this produced an unstable estimate of the association with ESCC risk. Meanwhile, those using non-solid fuel for heating appeared to have lower risk of ESCC, but this was also based on small numbers of participants. Thus, both of these associations were estimated for uncommon exposures, and should be interpreted with caution.

Among risk factors other than substance use, dietary consumption, or environmental exposures, HIV infection was present in 11% (n = 33) of cases and 4% (n = 12) of controls. HIV positivity was associated with ESCC (aOR 4.2, 95% CI: 1.9–9.4). Socioeconomic status (SES) score was not associated with ESCC (aOR 1.0, 95% CI 0.99–1.01). Subjects reporting that a sibling had died of esophageal cancer was associated with ESCC (aOR 2.5, 95% CI: 1.0–5.9, p = 0.04), but the number of subjects reporting this was limited, including only 8% (n = 24) of cases and 3% (n- = 10) of controls. Few subjects could report on esophageal cancer in their parents and there was no significant association with risk. A history of anemia and a history of hepatitis were not associated with ESCC, with aORs of 0.9 (95% CI: 0.3–2.2) and 0.2 (95% CI: 0.05–1.0), respectively.

## Discussion

Malawi has a high burden of ESCC [1], however the risk factors and causes are not well understood. In our study, we identified several risk factors to have a high risk for ESCC. Some are modifiable including smoking tobacco, drinking hot beverages and eating hot foods, consuming moldy grain, eating soil and HIV infection and some are non-modifiable like sibling history of ESCC.

Tobacco use has been associated with increased risk of esophageal cancer [12, 20, 23–25]. In our study we observed higher odds for self-rolled tobacco alone and for a combination of self-rolled and shop bought cigarettes, in concordance with other studies [23, 26–28] including previous reports from Malawi [29]. Self-rolled tobacco as smoked in Malawi has no filters and undergoes no processing. This could mean that smokers of this form of tobacco receive a higher dose of the carcinogenic products within the tobacco in comparison to the shop bought cigarettes. Similarly, access to self-rolled tobacco is easier among the smokers since Malawi is a major tobacco producer. Shop bought cigarettes alone and other forms of tobacco use did not have higher odds among all participants. However, the odds were non-significantly higher among all smokers who also drank alcohol, suggesting a synergistic relationship between the risk from tobacco and alcohol, as has been seen in other studies [20, 23, 24]. It is likely that the lack of statistical significance was due to the small number of subjects exposed to both tobacco and alcohol.

Alcohol use was not associated with increased odds of ESCC in our analyses. This is likely due to the small numbers of heavy drinkers (more than 3 drinks per day) in our dataset. In other populations, alcohol and ESCC risk have shown a dose-dependent relationship [20, 23, 24, 30, 31], but our population had few consumers in the range that typically shows significantly increased risk of ESCC. In addition, alcohol use is very minimal among females in our study population (only 2% of cases and 2% of controls), as reported in other studies [22], suggesting this exposure is not driving the high rates of ESCC in women in this population.

The link between hot food and beverages and increased risk for esophageal cancer has been reported by several studies [32–34]. Repeated thermal injury to the esophagus is thought to be the likely mechanism that leads to increased risk [35]. We did not see a temperature gradient elevation of risk, but this lack of effect must be interpreted with caution due to the lack of reliability of self-reported temperatures. Although our method for collecting tea temperature intake has been repeatedly used in other populations [13, 36], we have not independently validated our instruments in Malawi so it is possible that the lack of association was due to the data collection instrument or there could be a true lack of association in this region.

People who reported consuming moldy grain had a higher risk for ESCC, as has been previously reported and it is postulated that mycotoxins such as fumonisin B-1 in stored grain could be responsible for this increased risk [37–39]. These toxins are fairly common in maize samples in Malawi [40]. However, no direct causal link has been established [41]. Geophagia had a higher risk for ESCC, unlike what has been previously reported [15].

Low socioeconomic status of the participants did not confer higher risk. This might be due to matching by geographical region of origin, which most likely removed some variation in SES. Furthermore, we enrolled participants in a public and semi-public institution where the patients tend to be of a similar SES status.

Solid fuel use did not confer higher risk of ESCC, in contrast to what has been reported in other studies [16, 42]. In these other studies, the increased risk of ESCC was associated with solid fuel use in poorly ventilated rooms, where the generated smoke cannot escape and builds up in the room [42]. In contrast, the majority of Malawians cook outside or in well ventilated spaces [43, 44], and might therefore not have the same degree of exposure. The higher risk for ESCC among those reporting electricity use for cooking is probably a spurious finding, because the small numbers that used electricity led to unstable estimates.

Having a sibling with esophageal cancer conferred a higher risk for ESCC in our study, as has been previously reported [17, 45, 46]. However, because of the small numbers of cases and controls with affected siblings in our cohort, this difference should be interpreted with caution. We collected data on cause of death of parents to assess risk of ESCC. The estimates we produced were imprecise due to the small numbers reporting ESCC and also the large numbers reporting an unknown cause of death. We therefore excluded these results from the final analysis.

HIV positive status was associated with a higher OR for ESCC, as seen in previous studies [47]. Although the link between ESCC and HIV is not clearly understood, it has been postulated that higher co-infection of other oncogenic viruses among HIV positive individuals may lead to higher risk for developing ESCC [48, 49]. Other studies have also postulated that the increased risk among HIV positive participants may be due to the higher rate of alcohol intake and smoking among HIV positive participants [50, 51]. We found HIV positive status was associated with significantly increased risk of ESCC in models adjusted for tobacco and alcohol, so major confounding by those exposures should not explain the association we observed.

The strengths of the study include strong matching criteria, mobile-based data collection tools, and a histopathological confirmation of ESCC cases. Retrospective data collection and unmeasured confounding limit all case-controls studies. We also had a high proportion of participants with an unknown HIV status and the inability to analyze the effect of certain variables due to the matched study design. We reduced possible reporting bias by prior cognitive testing of the questionnaire and use of the same highly trained interviewers for all the participants to ensure that questions were asked in the same way. We also where possible used participant medical records, national identification cards and family members to get clarification and corroboration of collected data.

## Conclusion

Tobacco smoking, HIV infection, hot beverage and hot food consumption were observed to be risk factors for ESCC in Malawi. We further identified mold on stored grain and eating soil as risk factors. Our results are consistent with many findings from Africa and other regions. Many of the exposures that we identified as risk factors also increase the risk for other adverse health outcomes. Most of these risk factors are modifiable and can be addressed by public health messages to reduce the burden of ESCC and improve the overall health of this population.
